# Concurrent primary splenic lymphoma and mammary gland tumour with polycystic ovaries in a dog

**DOI:** 10.17221/99/2023-VETMED

**Published:** 2024-02-27

**Authors:** Seung-Hyun Kim, Yeong-Bin Baek, Sang-Ik Park

**Affiliations:** ^1^Laboratory of Veterinary Surgery, College of Veterinary Medicine, Chonnam National University, Gwangju, Republic of Korea; ^2^Laboratory of Veterinary Pathology, College of Veterinary Medicine, Chonnam National University, Gwangju, Republic of Korea; ^3^Laboratory of Veterinary Pathology, College of Veterinary Medicine, Chonnam National University, Gwangju, Republic of Korea; College of Veterinary Medicine and BK21 FOUR Program, Chonnam National University, Gwangju, Republic of Korea

**Keywords:** lymphoma, mammary gland tumors, myoepithelium, polycystic ovaries, surgical resection

## Abstract

Here, we report a rare case of concurrent primary splenic lymphoma and mammary gland tumour (MGT) with polycystic ovaries in a 10-year-old, intact female Jindo dog. The dog was presented with multiple masses in the fourth left mammary gland, the largest of which measured 6 cm in diameter, along with enlargement of the left inguinal lymph node on physical examination. Ultrasonography, radiography, and computed tomography scans revealed polycystic ovaries and a mass in the tail of the spleen, after total splenectomy and mastectomy with ovariohysterectomy, histopathological examination identified splenic diffuse large B cell lymphoma and malignant myoepithelioma of the mammary gland was found. To our knowledge, this is the first report of the concurrent occurrence of splenic lymphoma, MGT, and polycystic ovaries in a dog.

Canine mammary gland tumour (MGT) is the most common type of tumour in intact female dogs, with a malignancy rate of approximately 35–50% (Gilbertson et al. 1983). Interestingly, due to pluripotent stem-like cells in the mammary gland, canine MGT can develop various types of dysplastic and anaplastic cells (Rybicka and Krol 2016). The carcinogenic properties of MGT make its morphological classification challenging, although simple and complex carcinomas are the major types of MGT (Misdorp et al. 1999). Among the malignant forms of MGT, malignant myoepithelioma is a unique tumour of epithelial origin (Goldschmidt et al. 2011), comprising only a tiny proportion (0.1–2.2%) (Alonso-Diez et al. 2019). Moreover, diagnosing malignant myoepithelioma remains challenging due to a lack of information and limited number of cases.

Splenic masses are more common in older dogs, and nodular hyperplasia and hemangiosarcoma are the most common types (Cleveland and Casale 2016). Intriguingly, primary lymphoma derived from malignant lymphocytes within the spleen occurs rarely (0.02–0.1%) (Ito et al. 2014). According to the World Health Organization (WHO) system of human lymphoma, lymphoma classifications that are relevant to canine splenic lymphoma include marginal zone lymphoma (MZL), mantle cell lymphoma (MCL), diffuse large B cell lymphoma (DLBCL), follicular lymphoma, peripheral T cell lymphoma (not otherwise specified), and natural killer cell lymphoma (van Stee et al. 2015). Among these, diffuse DLBCL is the most common type and has a relatively high maturation and poor prognosis (Seelig et al. 2016).

Ovarian diseases in female dogs are uncommon but can lead to infertility and death. Among these, ovarian cyst is one of the most common types, whose classification is dependent on histopathological characteristics (Johnston et al. 2001), majorly including follicular cysts, cysts of subsurface epithelial structure, cystic rete ovarii, lutein cysts, and cystic corpora lutea (Knauf et al. 2018).

Here, using morphological classification and immunohistochemistry, we describe presumably the first case of concurrent primary splenic lymphoma and canine MGT, along with polycystic ovaries.

## Case presentation

**Figure 1:**
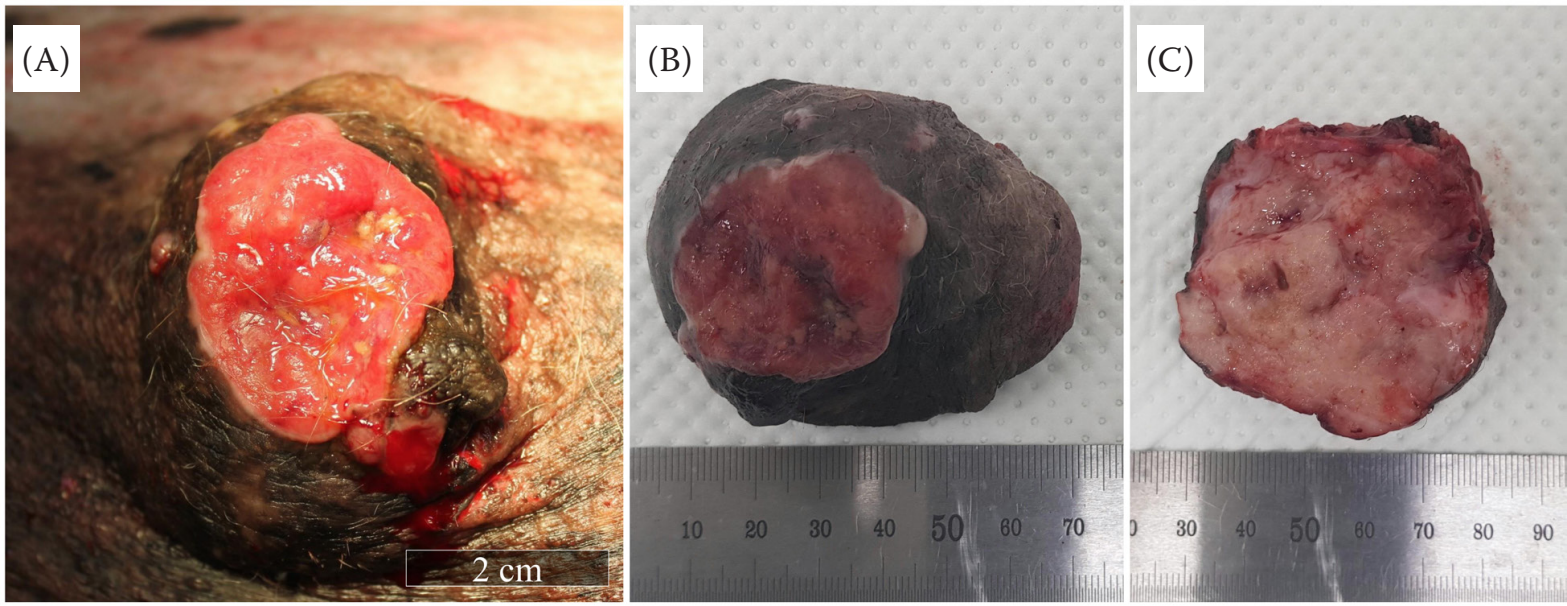
Gross examination of the largest mass in the fourth left mammary gland during (A) and after operation (B). The mass is presented on the cut surface (C) The mass measured approximately 6 cm in diameter, was firm in texture, and whitish in colour. Purulent exudate with central necrosis was observed, some of which leaked onto the surface

**Figure 2:**
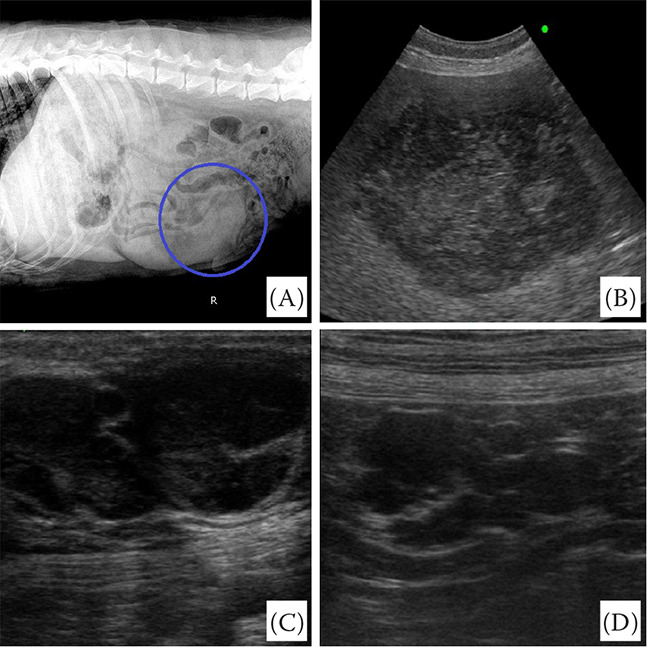
Abdominal radiography (A) and sonography (B–D) (A) A mass located on the spleen (circle). (B) Splenic incidentaloma. (C) Enlarged left inguinal lymph node. (D) Polycystic ovary on the left side

**Figure 3:**
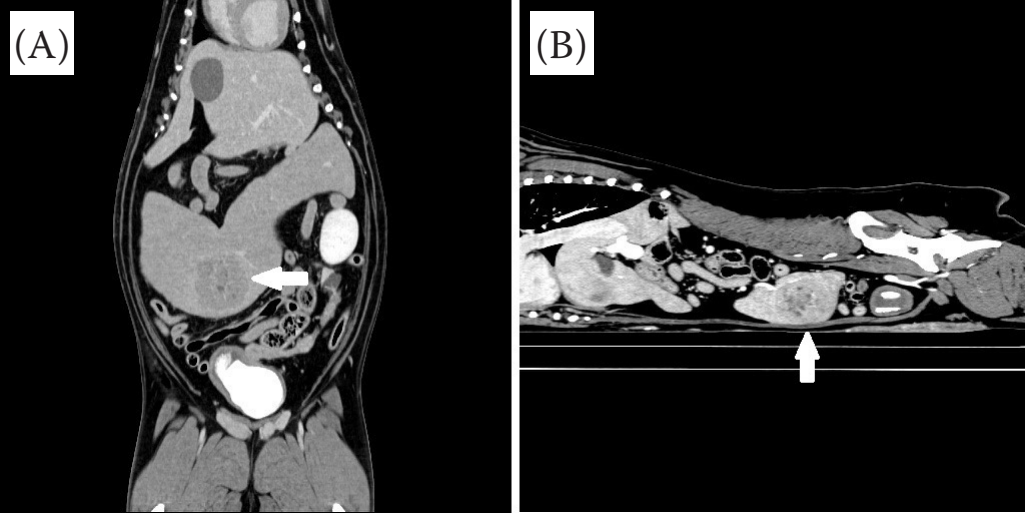
Abdominal computed tomography (CT) scan Coronal (A) and sagittal views (B) show a splenic incidentaloma (white arrow) located on the splenic body and displaying a low density

**Figure 4:**
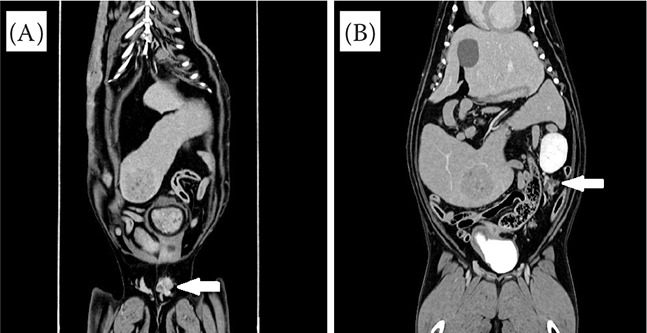
Abdominal CT scan (A) Enlarged left inguinal lymph node (arrow). (B) Left polycystic ovary (arrow)

A 10-year-old unneutered female Jindo dog presented with multiple masses in the fourth left mammary gland, which grew rapidly after the last oestrus. The largest mass was 6 cm in diameter and exhibited central necrosis with purulent exudate on the cut surface (Figure 1). Physical examination revealed enlargement of the left inguinal lymph node, suggesting a metastatic lesion. CBC revealed decreased MCV (54.1 fL) and MCH (18.5 pg) with increased RDW (24.1%) (Table 1), indicating microcytic anaemia. The results of the serum biochemistry test revealed increased levels of globulin (65 mg/ml), total protein (94 mg/ml), and CRP (18 μg/ml). Additionally, abdominal radiography and sonography detected multiple superficial masses, as well as incidentaloma at the tail of the spleen (Figure 2). In parallel, abdominal ultrasound (US) clearly showed an enlarged spleen with a mixed-echoic mass and multiple hypoechoic nodules located within the parenchyma. Round and enlarged left inguinal lymph nodes and bilateral polycystic ovaries were also detected. In the computed tomography, an expanding splenic mass was observed, showing a low-density lesion in the body region (Figure 3). Accordingly, enlargement of the left inguinal lymph node and bilateral cystic ovaries was also confirmed (Figure 4). Consequently, total splenectomy and mastectomy were performed along with OHE, which were subjected to fixation with 10% neutral formalin haematoxylin and eosin (H&E) staining or immunohistochemistry (IHC) were performed as previously described (Krenacs et al. 2010).

**Table 1:** Haematological and biochemical examination

Parameter	Results	Reference ranges	Conventional units
MCV	54.1 <	66–77	fL
MCH	18.5 <	21.0–26.2	pg
RDW	24.1 >	0–1.0	%
Globulin	65.0 >	27–44	mg/ml
Total protein	94.0 >	54–75	mg/ml
cCRP	18.0 >	0–10	μg/ml

cCRP = canine C-reactive protein; MCH = mean corpuscular haemoglobin; MCV = mean corpuscular volume; RDW = red cell distribution width

After washing in tap water, trimmed tissues were transferred to the automatic embedding machine for the following steps: gradual dehydration from 60% to 100% ethanol, clearance in xylene, and embedment in paraffin block. The paraffin block was cut at a thickness of 3 μm by a microtome, and H&E staining was performed. For IHC, the tissue section was rehydrated and autoclaved in 10 mM citrate solution (pH 8.0) for antigen retrieval. Subsequently, endogenous peroxidase activity was removed by treatment of 3% H_2_O_2_ for 30 min, and blocking with 5% bovine serum albumin was performed for 1 hour. The antigens were detected using primary antibodies (Table 2) and visualised by Dako REAL^TM^ EnVision^TM^ Detection System (Dako, Denmark) according to the manufacturer’s instructions.

**Table 2:** Primary antibodies used in immunohistochemistry

Antigen	Host	Dilution	Antigen retrieval	Source
Pancytokeratin	mouse	1 : 50	HIAR^a^	Dako, Glostrup, Denmark
Vimentin	rabbit	1 : 50	HIAR	Dako, Glostrup, Denmark
Smooth muscle actin	mouse	1 : 50	HIAR	Dako, Glostrup, Denmark
CD3	mouse	1 : 50	HIAR	Abcam, Cambridge, UK
CD20	rabbit	1 : 50	HIAR	Abcam, Cambridge, UK

^a^HIAR (heat-induced antigen retrieval) by autoclaving at 121 °C in 10 mM citrate buffer (pH 8.0) for 15 minutes

**Figure 5:**
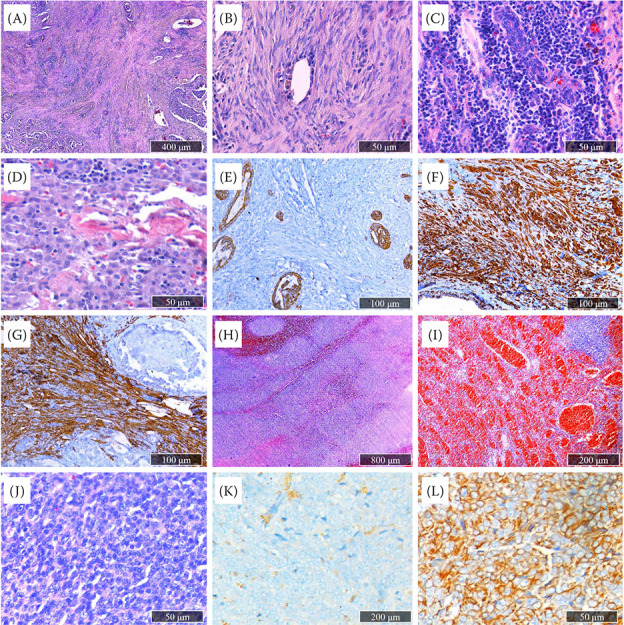
Histopathological characteristics of MGT (A–G) and splenic lymphoma (H–L) (A,B) Tumour lesions mainly consisted of fusiform myoepithelial cells with eosinophilic cytoplasm, showing moderate nuclear and cellular pleomorphism. (C) Some tumour cells differentiated into carcinoma lesions admixed with numerous of lymphoid cells. (D) Metastatic tumour lesion in the left inguinal lymph node. (E–G) Immunophenotyping revealed that tumour cells were negative for pancytokeratin (AE1/AE3) (E) and strongly positive for vimentin (F) and smooth muscle actin (G). (H,I) Neoplastic cells expand diffusely and aggressively, leading to loss of splenic architecture (H) and severe haemorrhage and necrosis (I). (J) Sheets of large centroblastic or immunoblastic B cells interspersed with tangible body macrophages. (K,L) Neoplastic lymphocytic cells were negative for CD3 (K) and positive for CD20 (L)

Microscopically, the mammary gland tumours, including the largest and smaller masses, were mainly composed of oval to spindle-shaped cells with a moderate amount of eosinophilic cytoplasm (Figure 5). The nuclei were longitudinal and central, with finely stippled chromatin and moderate nuclear pleomorphism. Some of this population produced variable basophilic fibrillary material (myxoid matric). Neoplastic cells forming tubular epithelium were often found admixed with macrophages, lymphocytes, and plasma cells. Moreover, metastatic tumour cells were found in the left inguinal lymph node. IHC confirmed that the main tumour lesions were negative for pan-cytokeratin but highly positive for vimentin and smooth muscle actin, strongly indicating a diagnosis of malignant myoepithelioma.

In the spleen, tumour lesions expanded diffusely and destroyed the normal architectures, resulting in locally extensive haemorrhage and necroses (Figure 5). Neoplastic cells were large (2 times bigger than RBC), and their nuclei were variable in shape, forming large sheets with interspersed tangible bodies of macrophages. According to immunophenotype, the main lesions were negative for CD3 and positive for CD20. Centroblasts and round, large immunoblasts had euchromatic nuclei with vesicular or coarsely granular chromatin, which were multiple in centroblasts but were solitary in the center in immunoblasts. Therefore, these lesions highly indicated a diagnosis of DLBCL.

## DISCUSSION

Canine MGT is the most common type of tumour in intact female dogs, and its carcinogenesis is highly linked to oestrogens. In bitches, preventive OHE performed in the early stage of life significantly reduces the incidence of MGT (Canadas-Sousa et al. 2019). Unlike human breast cancer, anti-oestrogen therapy, including agents such as anti-aromatases, selective-oestrogen–receptor-modulators, and oestrogen-blockers, is contraindicated in canine MGT due to significant side effects such as pyometra (Gray et al. 2020). Therefore, the current mainstream treatment option for canine MGT is surgical resection, except for inflammatory mammary carcinomas, including simple mastectomy, partial mastectomy, and total mastectomy, along with simultaneous OHE (Sleeckx et al. 2011). Even when the metastatic lesion is confirmed, it can induce clinical symptom re-lief, increase survival time, and lower recurrence and metastasis (Hornfeldt and  Mortensen 2023). In human medicine, postoperative adjuvant chemotherapy has increased survival rates (Karayannopoulou et al. 2001). However, postoperative systemic chemotherapy is not applicable in canine MGT due to the absence of a standardised protocol (Simon et al. 2006). Only a few studies have reported that chemotherapy reduced recurrence and prolonged survival (Simon et al. 2006), necessitating further research on postoperative adjuvant chemotherapy in canine MGT.

Notably, ovarian cysts can represent a significant source of oestrogen, leading to hyperoestrogenism, which possibly induces gynaecological diseases of the uterine and mammary glands (Johnston et al. 2001; Knauf et al. 2018). Although this case did not show any significant clinical signs, hyperoestrogenism due to bilateral polycystic ovaries could theoretically give rise to the pathogenesis of the primary development of MGT and subsequent metastasis in an adjacent lymph node.

In splenic lymphoma, preventive splenectomy is preferred because of the risk of splenic rupture during the exploration of diagnosis, which can also be applied for pain management and unresponsiveness to chemotherapy (van Stee et al. 2015). Without postoperative adjuvant chemotherapy, splenectomy alone can induce complete remission. Meanwhile, chemotherapy may not prolong the survival time in dogs (O’Brien et al. 2013; Sabattini et al. 2018), implying that it is not a good treatment option for the patient. Therefore, splenectomy is the treatment of choice, corresponding with the treatment protocol of human DLBCL (van Stee et al. 2015).

Here, presumably for the first time, we describe a case of simultaneous multiple tumours in an intact female dog, including splenic lymphoma and metastatic MGT, together with polycystic ovaries. Although complete resections were achieved for each disease, we failed to proceed with further treatment using chemotherapeutic agents. The patient was doing well without any signs of recurrence or distant metastasis for over three years, confirmed by periodic physical examination, radiography, and sonography. It is a very encouraging result considering that the average 1-year survival rate of patients with lymphatic metastatic MGT is 19% and that of patients with splenic lymphoma is 69.8%, respectively (van Stee et al. 2015; Rasotto et al. 2017). This result can be of clinical significance in that it led to treating concurrent tumours through complete surgical resection of each neoplasm. Since then, we have not reached the patient due to loss of contact. For the following study, the further clinical application will be necessary for MGT patients to develop standardised adjuvant chemotherapy.
